# JACMP 2020–2024

**DOI:** 10.1002/acm2.14447

**Published:** 2024-06-30

**Authors:** Susan L. Richardson

**Affiliations:** ^1^ Swedish Cancer Institute Radiation Oncology Seattle Washington USA

## 1 MY INTRODUCTION TO THE JACMP

I first met Mike Mills in Kentucky in the mid‐2000s. He was one of my ABR oral examiners, and a kind and respectful one even when I blanked on what a klystron was called … luckily, I managed to pass the exam despite my memory lapse. I bumped into him at a meeting a few years later and jokingly thanked him for his empathy and for passing me. I don't think he remembered me at the time although he said he did. We started to talk, and of course, if you talk to Mike, you talk about the JACMP. He pulled me into his figurative circle and thus began my involvement with the JACMP, first as a reviewer, and later as an associate editor and member of the board of editors.

Like many AAPM members, I knew the journals were an important component of our organization, but it wasn't until I was a member of the executive committee that many things clicked, particularly regarding the importance of the JACMP. An overwhelming percentage of the AAPM is clinical, non‐academic physicists who rely on well‐written reports, clinical guidelines, and open‐access environments to learn from others about best practices. The articles published in JACMP are clinically relevant and directly impact patient care. The JACMP provides all of these not only to AAPM members, but to the whole world. Developing countries may not have access to other scientific journals with paywalls or subscription fees. The small profit the AAPM makes while publishing the JACMP seems like a win‐win for both the AAPM and the patients and physicists worldwide because of the access and education it provides.

When Mike called me in late 2021 and asked me if I would consider being a deputy editor‐in‐chief, I felt honored and privileged to accept the offer and help support the journal that contributes so much to the medical physics landscape. While I only have held this position briefly, I am happy to share the following editorial regarding how I view the JACMP today.

## 2 THE CHANGING LANDSCAPE OF THE JACMP

**FIGURE 1:**
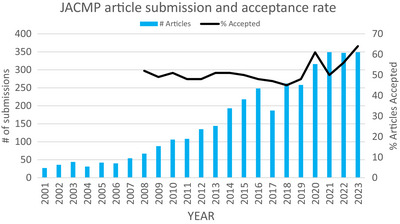
JACMP article submission and acceptance rate.

The year 2020 hit most of us hard. With the onset of COVID‐19, we were struck emotionally, physically, and scientifically—research projects were halted; and meetings were canceled. Hospital and clinical staff were sent home, conscripted, or laid off. What did that mean for the JACMP? As shown in Figure 1, the JACMP was surprisingly untouched by covid with almost 60 more submitted articles in 2020 than in 2019. Ironically, COVID‐19 also provided an opportunity for practitioners to share problems and solutions related to COVID‐19. One of the most downloaded articles of all time for the JACMP is Mary Beth Allen's “How has the COVID‐19 pandemic changed patient care and the practice of medical physics in an academic environment?^1^” Even in 2023 a COVID‐19 article remains among the top 10 downloaded articles of the year (Table 1). The parallel opposed article regarding medical physicists working from home was a great help for members trying to balance work and personal needs during this challenging time.^2^ I also read with great interest the article regarding the in‐person AAPM meeting being a superspreader event—an event that I attended but remained healthy through.^3^

**TABLE 1:** Wiley library's most downloaded articles in 2023.

Most downloaded articles in JACMP in 2023					
Rank	MPPG	Authors	Topic/title	Year	No. of downloads
1	✓	Smilowitz, et al.	MPPG 5.a: Commissioning TPS algorithms	2015	10059
2	✓	Smith, et al.	MPPG 8.a: Linac performance tests	2017	9942
3	✓	Halvorsen, et al.	MPPG 9.a: SRS‐SBRT	2017	8022
4		Mary Beth Allen	COVID‐19	2022	7209
5	✓	Xia, et al.	MPPG 11.a: plan and chart review	2021	6448
6	✓	Richardson, et al.	MPPG 13.a: HDR brachytherapy	2023	6410
7	✓	Guerts, et al.	MPPG 5.b. Commissioning TPS algorithms	2023	5759
8	✓	Krauss, et al.	MPPG 8.b Linac performance tests	2023	5186
9	✓	Fisher, et al.	MPPG 12.a: Fluoroscopy dose management	2022	4756
10		Hu, et al.	Medical image analysis	2023	4753

Moving on from the pandemic, the next topic that seems huge for the JACMP is the publication of Medical Physics Practice Guidelines (MPPGs). While Task Group reports are typically still published in Medical Physics, the JACMP has been the home for MPPGs. The first MPPG was published in 2013, but since then there have been a total of 15 MPPGs published, including revisions. Five MPPGs were published in 2023 alone. The topics included are clinical, diagnostic, and professional, branching through all subfields of medical physics. The MPPGs are the most downloaded articles from the JACMP, claiming 8 of the top 10 slots in 2023. Of note, while the number of downloads is high, the citation rate of these articles is rather low, indicating that they are practical and useful, but not often quoted in scientific literature. This is an important distinction for the JACMP as the JACMP showcases publications requiring a different metric of value rather than just typical citations and impact factors.^4^

*Note*: The early publications from the early 2020s showcased a variety of new technology and techniques. These include MRI linacs, ring gantries, and automated QA techniques. It also was the time of the explosion on topics of deep learning, artificial intelligence, and automation.

## 3 MODERN CHALLENGES TO THE JACMP

With the rise of the submission on topics of AI, so rose the challenges in publishing and journal rectitude. The use of ChatGPT and other forms of AI in both ethical and unethical article creation increases the hurdles placed on the humans that support journal production. It becomes more important to evaluate not only the submitted content but how the content was generated and the data obtained. Some tools, such as IThenticate (which is used by Wiley), help to evaluate the overlap of a submission with existing publications, but it is not without loopholes. iThenticate is susceptible to articles that have been rewritten by AI specifically to bypass gatekeeping plagiarism tools. Additionally, iThenticate does not validate or cross‐check images, which are easy to replicate from online sources.

Expanded adoption of AI tools increases the difficulty in establishing misuse. The burden of assessing if an article is valid, original, and human written, lies squarely on the shoulders of the editors and the reviewers. As described in a blog post by Priya Madina, Director of External Affairs and Policy at Taylor & Francis, “*AI offers unprecedented opportunities for equity, accessibility, and combating misinformation and disinformation in academic publishing, the technology also presents significant challenges to the integrity and legitimacy of scholarly content*.^5^ *”* This problem has undoubtedly reached the JACMP and the medical physics community. One article recently submitted to the JACMP contained the exact same data and figures as a previously published (non‐JACMP) article. It was discovered at the editorial level mostly due to diligence of the editor; it was not flagged by iThenticate as the text of the article had been rewritten (possibly by AI) and the figures were not assessed by this tool thus by‐passing any detection from this mechanism.

We have all used spell‐check and asked the paperclip to review our writing. Many authors adopt smart tools such as Grammarly to edit and increase readability of our publications. But somewhere is a line between receiving assistance from AI tools and allowing AI to dictate the content and tone of the paper. It is a colloquial grey area, and the line moves from year to year as more AI tools are adopted and become commonplace. Treading this proverbial line in a fair and thoughtful manner that promotes scientific integrity will challenge the reviewers and editors of the JACMP for years to come.

One step beyond using AI to write a paper is allowing someone else or another entity to generate the publication entirely. Papermills produce and sell fraudulent manuscripts masquerading as legitimate research. The Committee on Publication Ethics (COPE) investigated this phenomenon in academic publishing and created an excellent report.^6^ These papermill documents may contain fictitious or manipulated data or plagiarize other valid work. Authors may purchase entire documents to submit or purchase partial authorship in a submission. One publisher analyzed by COPE and STM summarized that the percentage of affected articles in each journal published or submitted between 2019 and 2021 was an astounding 14% (range 2%−46%).

Combating unethical publications is Retraction Watch (https://retractionwatch.com/), a highly established blog that provides a database identifying which journals are at high risk of being duped and a retracted author and publication list. While the journals at the highest risk tend to be those in the biochemistry and genetics fields, medical physics is not immune. JACMP has one retracted article listed in the database. Resources such as the ones provided in Retraction Watch are critical in combatting unethical submissions resulting from the lack of communication and shared platforms between journals. Recently, I invited an Associate Editor (AE) to review a seemingly valid article at the JACMP. The AE notified me that they were already reviewing this *exact article* in a *different* journal. Without the sheer luck of picking that same subject expert, the paper might have been accepted and published on both platforms. While there is no evidence that the article came from a papermill, it may be reasonable to assume that someone so desperate to publish might just purchase an article and submit it to multiple places. While the authors can be banned from JACMP and Med Phys following COPE guidelines, there is nothing to stop the authors from submitting to another, independent journal, and no way to notify those journals of the inappropriate submission.

There is one more threat that all journals must be on the lookout for and that is journal hijacking. Hijacked journals are website spoofs of legitimate journal websites that lure in submissions. Hackers may copy the target ISSN (the publisher's identification number), title, and even steal photo headers. The websites appear to be authentic journal submission website portals. The hackers will then solicit for and steal the submission and/or publication fees, and even possibly the article itself. As described in a Clarviate blog, the hijackers may pay fees to Google to become the first hit when using that search engine for that journal. In the example described on the website, the legitimate journal was actually the third link down in the search engine results.^7^ In this example, the spoofed website was for an Icelandic earth sciences journal, however, the landing page showed a picture of Mt. Rainier, which I can see from my building in Seattle, Washington.

## 4 THE NEXT FEW YEARS

The JACMP remains committed to equity, diversity, and inclusion initiatives and will continue to do so for the future. A previous editorial has illustrated how important the double‐blind system of the JACMP is and the many ways in which the JACMP challenges conventional publication models.^8^ The international reach and open access model removes many barriers to accessing important scientific guidance. The waivers of publication charges by JACMP also assist authors who may not be able to afford the fees in US dollars. The JACMP will continue to be a leader in Equity, Diversity, and Inclusion (EDI) and an early adopter of many enterprises that support our readers and authors, such as signing on to Declaration on Research Assessment (DORA).

## 5 CONCLUSIONS FOR 2024

The challenges facing the JACMP and journals, in general, are complex and difficult to solve with a single wide brush stroke. While the number of submissions continues to increase, the care and time required to evaluate, review, and assess each submission rises as well. Much of the evaluation is still manual and must be done by humans. As the editorial staff remains small and reviewers uncompensated at this point, we are left to volunteerism and altruism to perform much of the work to ensure high standards of scientific integrity. That is not to say we are unsupported, on the contrary, Wiley is now testing an enhanced and upgraded version of iThenticate, designed to help editorial staff evaluate publications to a higher level than before. But as we adapt, so do *they*, and we must keep a constant vigil to keep the JACMP on its upward trajectory.

## CONFLICT OF INTEREST STATEMENT

The author declares no conflicts of interest.
